# A Randomised, Double-Blind, Placebo-Controlled, Cross-Over Clinical Trial to Evaluate the Biological Effects and Safety of a Polyphenol Supplement on Healthy Ageing

**DOI:** 10.3390/antiox13080995

**Published:** 2024-08-17

**Authors:** Joyce Ruifen Chong, Chiara de Lucia, Diego Alejandro Tovar-Rios, Nicolas Castellanos-Perilla, Christopher Collins, Silje Meihack Kvernberg, Clive Ballard, Richard C. Siow, Dag Aarsland

**Affiliations:** 1 Centre for Healthy Brain Ageing, Institute of Psychiatry, Psychology and Neuroscience, King’s College London, London S5 9NU, UK; dag.aarsland@kcl.ac.uk; 2 Department of Pharmacology, Yong Loo Lin School of Medicine, National University of Singapore, Singapore 117600, Singapore; 3 Centre for Age-Related Medicine, Stavanger University Hospital, 4019 Stavanger, Norway; diego.tovar@correounivalle.edu.co (D.A.T.-R.); nicolas.castellanos.perilla@ki.se (N.C.-P.); silje.meihack.kvernberg@sus.no (S.M.K.); 4 Grupos de Investigación en Estadística Aplicada—INFERIR, Faculty of Engineering, Universidad del Valle, Santiago de Cali 760042, Colombia; 5 Grupo Prevención y Control de la Enfermedad Crónica—PRECEC, Faculty of Health, Universidad del Valle, Santiago de Cali 760042, Colombia; 6 Department of Clinical Medicine, University of Bergen, 5009 Bergen, Norway; 7 Semillero de Neurociencias y Envejecimiento, Ageing Institute, Medical School, Pontificia Universidad Javeriana, Bogotá 110231, Colombia; 8 Department of Neurobiology, Care Sciences and Society, Karolinska Institutet, 171 65 Stockholm, Sweden; 9 Muhdo Health Ltd., Columba House, Adastral Park, Martlesham Heath, Martlesham, Ipswich IP5 3RE, UK; chris.collins@muhdo.com; 10 University of Exeter Medical School, University of Exeter, Exeter EX1 2HZ, UK; c.ballard@exeter.ac.uk; 11 School of Cardiovascular and Metabolic Medicine & Sciences, British Heart Foundation Centre of Research Excellence, Faculty of Life Sciences & Medicine, King’s College London, London SE1 9NH, UK; richard.siow@kcl.ac.uk; 12 Ageing Research at King’s (ARK), King’s College London, London SE1 9NH, UK; 13 Department of Physiology, Anatomy and Genetics, Medical Sciences Division, University of Oxford, Oxford OX1 3PT, UK

**Keywords:** oxidative stress, inflammation, mitochondrial dysfunction, Mediterranean diet, DNA methylation

## Abstract

DailyColors™ is a supplement made up of several phytonutrients that aims to replicate elements from the Mediterranean diet. These include fruit, berry and vegetable extracts that are rich in key phytochemicals such as Quercetin, Catechins, Phloretin, Ellagic Acid, and Anthocyanins. Here, we determined the effects of DailyColors™ on the blood biomarkers associated with the diverse mechanisms implicated in ageing and age-related diseases, including mitochondrial function, inflammation, and oxidative stress, as well as on saliva’s DNA methylation pattern. Thirty adult participants (mean (SD) age = 67.0 (7.5) years) with a body mass index over 25 were recruited into this randomised, double-blind, placebo-controlled, cross-over trial (two one-week treatment periods, separated by a one-week washout period). During the placebo period, we observed a significant increase in blood CD38 concentrations from the baseline to 24 h (*p*-value = 0.019). This was not observed in the active period. Increased CD38 is reportedly associated with subsequent mitochondrial dysfunction and inflammation. Next, there was a decreasing trend of plasma 4-HNE levels, an oxidative stress biomarker, after a one-week intake of DailyColors™. Furthermore, following a one-month open-label follow-up in 26 participants, we observed hypermethylation of the candidate CpG site cg13108341 (q-value = 0.021), which was against the observed trend for this site during ageing. Taken together, while minimal effects were observed in this study, DailyColors™ supplementation may be beneficial by altering and alleviating age-related changes. Longer and larger scale trials of DailyColors™ supplementation are warranted.

## 1. Introduction

With increasing life expectancy, attention and interest in safe and effective lifestyle interventions to promote healthy ageing are growing rapidly. Accumulating evidence suggests that modifiable vascular risk factors such as hypertension and diabetes, as well as lifestyle factors such as physical inactivity and unhealthy diets (with poor nutritional value), are associated with an increased risk of developing age-related diseases such as dementia [1]. Diverse molecular mechanisms are implicated in ageing and in the pathogenesis of age-related diseases, such as increased reactive oxygen species production and oxidative stress, chronic inflammation, abnormal protein aggregation and accumulation, mitochondrial dysfunction, and changes in the DNA methylation pattern [2,3,4]. In this regard, it is postulated that lifestyle changes, including dietary interventions that promote anti-inflammatory and antioxidant effects, as well as the modulation of DNA methylation, could potentially reduce the risk of developing ageing-related diseases [3,5].

Previous studies showed that phytonutrients, which are the natural compounds produced by plants, such as vegetables, fruits, and berries, support health. The Mediterranean (MeDi) diet, a dietary pattern followed by the Mediterranean Basin countries, is mainly based on abundant phytonutrients. Briefly, the MeDi diet consists of olive oil as the primary source of fat, fresh fruit, and low-to-moderate amounts of fish, seafood, poultry, dairy products, wine, and eggs. Sweets containing sugar or honey and red meat are consumed sparingly. Recent systematic reviews and meta-analyses reported that adherence to the MeDi diet was associated with a lower risk of age-related conditions such as cardiovascular diseases [6,7,8] and dementia [7,9,10], suggesting adherence to this diet may be protective against several conditions. 

DailyColors™, a supplement that includes elements from the MeDi diet in a capsule, blends ingredients from 16 fruits, vegetables, and herbs from the MeDi diet and contains over 150 highly bio-active compounds isolated from the red, blue, green, and orange colours in these plants. However, the effects of DailyColors™ on the key processes implicated in ageing remain largely unknown. We hypothesise that the consumption of DailyColors™ for a short period of one week positively affects the biological pathways related to healthy ageing, such as mitochondrial dysfunction, inflammation, oxidative stress, and epigenetics. 

In this exploratory study, we aimed to assess the effects of DailyColors™ on the blood biomarkers associated with mitochondrial function [cluster of differentiation 38 (CD38) and nicotinamide adenine dinucleotide (NAD+)], inflammation [tumour necrosis factor (TNF)-α and interleukin (IL)-6, IL-8, and IL-10], oxidative stress [4-hydroxynonenal (HNE), protein carbonyl, and total antioxidant capacity (TAC)]. In a secondary analysis, we also aimed to assess the effect of the intervention on DNA methylation and to assess the safety of DailyColors™. Importantly, our intervention targeted individuals with a body mass index (BMI) over 25, as these individuals are more likely to exhibit disrupted metabolism and inflammation [11] and respond to the beneficial effects of the supplement. 

## 2. Materials and Methods

### 2.1. Study Design 

**Figure 1:**
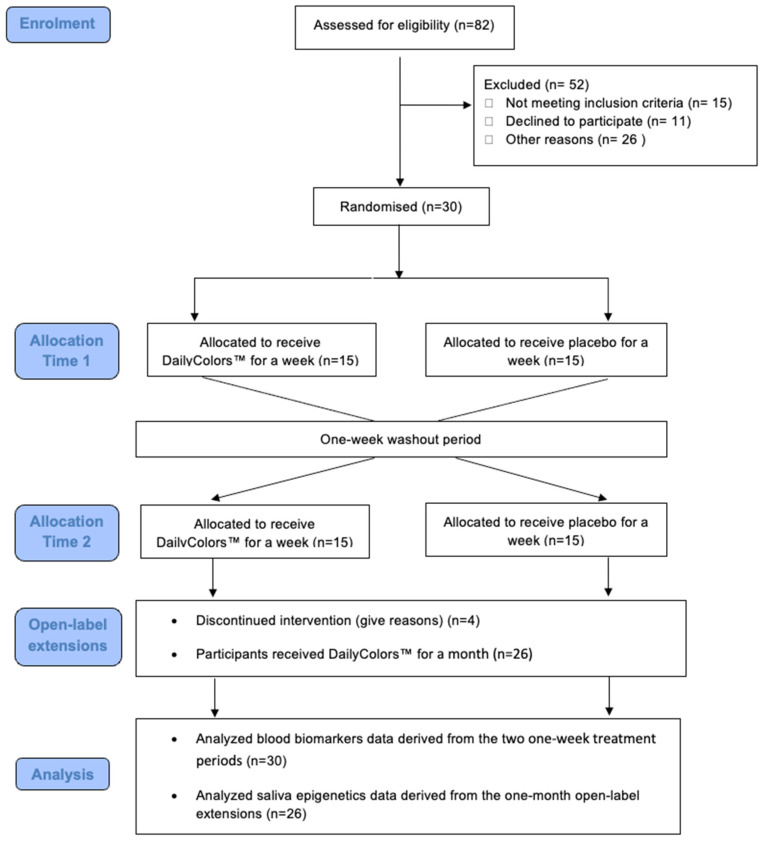
Flow diagram illustrating the study design.

This is a randomised, double-blind, placebo-controlled cross-over trial (Figure 1) that took place in Norway between February and June 2023. The placebo-controlled trial was followed by a one-month open-label period where all the participants received the active treatment. The recruitment involved Norway’s Stavanger region through clinical settings, previous research cohorts, social media, and traditional media outlets. The interested individuals were provided with study information and contact details for the research team. Thirty participants were recruited from the public. A brief assessment by a medical doctor included history taking, drug use, and a physical examination. Routine blood tests were performed to exclude relevant medical conditions. Vital signs were measured, including waist circumference, heart rate, and blood pressure. Weight and height were measured, and the BMI was calculated. All the participants provided written informed consent. This study was approved by the Regional Ethics Committee in Western Norway and the Research Director at Stavanger University Hospital and registered at ClinGov NCT05829382.

### 2.2. Eligibility Criteria

This study recruited participants who were 55 to 80 years of age and willing to complete the questionnaires, records, and diaries associated with this study and all clinic visits. Furthermore, the participants had to have a body mass index (BMI) over 25. The exclusion criteria included alcohol or drug abuse in the past year; a known allergy to the test material’s active or inactive ingredients; unstable or life-threatening medical conditions; the use of polyphenol or phytochemical supplements; the use of dietary supplements in the last month, except for the normal intake of cod liver oil or other vitamin D supplements; vegetarians and vegans; clinically significant abnormal laboratory results at screening; participation in a clinical research trial within 30 days before randomisation; dementia or an inability to give informed consent; an unusually high fruit, berry, vegetable, or coffee intake (e.g., >five portions of fruit, berries, or vegetables per day or >five cups of coffee daily); or any other condition that, in the investigator’s opinion, might adversely affect the subject’s ability to complete this study or its measures or pose a significant risk to the subject.

### 2.3. The Intervention

DailyColors™ capsules contain 150 mg of the phytonutrients listed in Supplementary Table S1. Further details are available in the Daily Colors US Patent Application (serial no. 17/843,140), which also contains the details of the phytochemical HPLC/MS/MS analyses. The daily dose for both the cross-over trial and the open-label extension consisted of one DailyColors™ capsule. Microcrystalline cellulose was used in the placebo capsule, produced with a similar appearance as the active compound visually and regarding smell and taste. The treatments were randomly assigned using a computer to each participant in two sequences: receiving DailyColors™ once daily for a week, followed by a one-week washout, then a placebo for a week (“Active/Placebo”) or the opposite order, e.g., one week of a placebo, the washout, and one week of DailyColors™ (“Placebo/Active”). Following the cross-over intervention, the participants were invited to a one-month open-label continued intake of DailyColors™ once daily for a month, which started 3 to 8 weeks after the cross-over intervention ended. The participants were requested to avoid significant lifestyle changes during the study period. 

### 2.4. Procedures

For the cross-over intervention, the participants were asked to come to the clinic after fasting for 12 h for each clinic visit. At the baseline visit for each treatment period, the participants took the first capsule after the first blood collection (T0), had a standardised meal, and then completed blood draws one (T1) and two (T2) hours after taking the first capsule. They came, after fasting, for the 24 h visit and took the next capsule after the 24 h (T24) blood draw. At the final visit (D7), the participants again came after fasting, but they had taken their last capsule as usual before arriving at the clinic. At each visit, their vital signs (i.e., heart rate and blood pressure) were measured; concomitant therapies were reviewed. For the open-label extension, the participants were asked to consume daily DailyColors™ capsules for one month. Saliva samples were collected prior to the consumption of the first capsule (T0) and following the end of the open-label extension (V6). Compliance was assessed by counting the returned capsules.

### 2.5. Blinding

To ensure blinding in this study, a designated guardian conducted the random treatment allocation by generating random numbers in Excel and securely held this information, keeping it undisclosed to the laboratory researchers who performed the blood biomarker measurements, as well as to the statistician, until after the analyses. The statistician only had access to anonymised treatment labels throughout this study.

### 2.6. Outcome Measures: Blood Biomarker Assessments

Blood was drawn from the study participants into ethylenediaminetetraacetic acid (EDTA) or CAT Serum Separator tubes and processed by centrifugation at 1200× *g* for 10 min at room temperature, followed by the extraction of the plasma and serum layers, respectively. The plasma, serum, and whole blood samples were stored at −80 °C until use. 

All the target analytes were measured in duplicates using commercially available kits, according to the manufacturer’s protocols. The NAD+ levels were measured using the Q-NADMED Blood NAD+ assay kit (NADMED Ltd., Helsinki, Finland), which allows the quantification of NAD+ in whole blood. The plasma CD38 levels were measured using the Abcam’s Human CD38 ELISA kit (Abcam Limited, Waltham, MA, USA). The NAD+ and CD38 levels were considered the primary outcomes. The cytokines levels in the serum were measured using the Luminex platform. We used the customised MILLIPLEX^®^ multiplex assays from Merck Life Science (Merck KGaA, Darmstadt, Germany). For the cytokines, some measurements were below the detection limits and, as such, replaced by the minimum detectable concentrations: IL-6, 0.18 pg/mL; IL-8, 0.24 pg/mL; IL-10, 0.72 pg/mL; and TNF-α, 4.40 pg/mL. The plasma’s total antioxidant capacity (TAC) levels were measured using the Abcam’s Total Antioxidant Capacity Assay Kit (Abcam Limited, Waltham, MA, USA). The plasma’s 4-HNE and protein carbonyl levels were measured using ELISA kits from Cusabio (Human 4-Hydroxynonenal ELISA Kit, CUSABIO TECHNOLOGY LLC, Houston, TX, USA) and Abcam (Protein Carbonyl ELISA Kit; Abcam Limited, Waltham, MA, USA), respectively. All the target analytes were measured at T0 (the baseline) and at the end of each 1-week study period (D7). In addition, NAD+ was measured 1 (T1), 2 (T2), and 24 (T24) hours after the intake of the first capsule in each period. CD38 was also measured 24 h (T24) after the first capsule intake.

### 2.7. Outcome Measures: Epigenetics

The epigenetic analysis focused on the saliva samples obtained from the trial participants at T0 before the first intake and visit 6 (V6) following the end of this open-label study. Therefore, all the participants received 5 weeks of active intervention over the total study period. The saliva was collected using standard saliva collection kits (Isohelix GFX-02, Cell Projects LTD., Harrietsham, UK), and a CpG site methylation analysis was performed commercially (Eurofins, Galten, Denmark) using the Illumina Infinium MethylationEPIC 850k genome-wide BeadChip array (Illumina, CA, USA).

The MethylPace clock (Muhdo Health, Ipswich, UK) was created using a preexisting cohort of 4000 individuals. Within this cohort, 70% had 850,000+ CpG/SNP sites analysed via the Illumina EPIC 850k methylation test, while 30% had 30,000 CpG/SNP sites analysed via an Illumina MASKED array. Furthermore, many subsequent tests (i.e., subjects with more than one analysis) were tested via the 30,000 CpG/SNP site MASKED array. 

Though the MethylPace clock consists of 3 analysis layers, in this paper, we only used the first of these, given the limited number of participants. Layer one focuses on 9 CpG sites that have shown a tight correlation to ageing, meaning these 9 sites can predict an individual’s age. This specific combination of CpG sites was selected via Muhdo’s internal data analysis following findings showing high correlations to epigenetic age [10,11,12,13]. The 9 CpG sites included in this analysis are shown in Table 1. As standard, the results are presented as beta values, which describe the frequency of DNA methylation at a given CpG site. Following this targeted analysis, the differences in DNA methylation across all the measurement sites were investigated. The top 1000 sites showing differential DNA methylation were recorded.

### 2.8. Statistical Analysis

Sample size analysis: This exploratory study was not powered to detect statistically significant differences but rather aimed to identify potential impacts, initiate the understanding of potential mechanistic pathways, and inform further study designs. We used available in-house data for the sample size calculations. These were based on a small group of healthy adult participants, with hourly measurements of red blood cell NAD+ concentrations by total protein after the intake of the DailyColors™ capsules (150 mg) or the placebo. We observed significant differences between the active and placebo groups, with an effect size of 0.46. Based on this, a minimum sample size of 20 participants would detect an effect size of 0.46 with a statistical power exceeding 90%. To account for potential dropouts, we recruited 30 participants, ensuring a sufficient number of participants to provide a full set of blood samples for analysis.

Baseline descriptive analysis: The descriptive analysis of the baseline data included calculating the means and standard deviations for the quantitative variables and the percentages for the categorical variables.

Exploration of treatment effect on blood markers: To investigate the treatment effect, a longitudinal mixed model was employed. The model included factors for time points and treatment, as well as their interaction. The model incorporated a binary variable for the treatment period and an indicator for the treatment change to account for the carry-over effect after the washout period. Additionally, an overall subject random intercept by period was included in the model to control for the variability and correlation arising from repeated measurements. We examined the potential influence of sex, age, and BMI as the control factors. Among these, BMI was found to be significant only in the case of IL-6, while sex was significant for TNF-α. We explored the contribution of age as a continuous and categorical variable (<65), but age did not contribute significantly to any of the models. The models were fitted using the restricted maximum likelihood (REML) method. The hypothesis testing for the least squared means post-estimations employed the Kenward–Roger method to estimate the degrees of freedom. To compare the treatments at each time point, contrasts were utilised. Because of the multiple testing performed, the false discovery rate (FDR) correction-adjusted *p*-values were applied. We additionally performed the analyses by excluding those with high coefficients of variation (CoVs) and any outliers, after excluding two participants who did not fast at one of the baseline assessments. A high CoV was defined as a CoV ≥ 25%. Outliers were defined as observations with a standardised residual ≥ 2.5 (2.5 standard deviations). Hypotheses were rejected based on an alpha level of 0.05. All the data analyses and graphics were performed using R version 4.2.1.

Exploration of supplementation change on epigenetics: Twenty-six participants who took part in the open-label extension were included in this analysis. The primary focus of this exploratory analysis was to assess the statistical significance of the changes observed in the nine selected CpG sites before (T0) and after (V6) the supplement intake. Paired *t*-tests were employed for comparison, with the *p*-values adjusted for multiple testing using the FDR method.

## 3. Results

### 3.1. Study Cohort

Thirty participants who fulfilled the inclusion and exclusion criteria were recruited and randomised into the cross-over trial. Of these 30 participants, 26 participants continued with a daily intake of DailyColors™ for a further month as part of this open-label study. The overall compliance was high, with >90% of the participants adhering to the planned schedule and study protocol. Only two participants failed to fast at one of the baseline assessments. The baseline participants’ characteristics are shown in Table 2. The mean (SD) age was 67.0 (7.5) years, 53.3% of the participants were female, and the BMI was 28.7 (3.0) kg/m^2^.

### 3.2. Outcomes: Blood Biomarker Assessments

**Figure 2:**
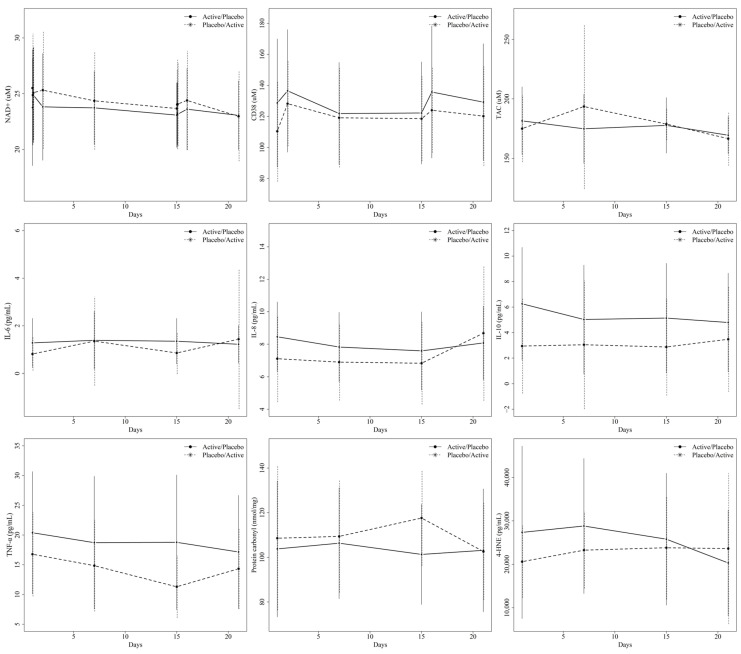
Graphs illustrating the means and standard deviations for each biomarker concentration at each time point in the two groups (active/placebo or placebo/active sequences). The time between days 6 and 14 represents the washout period.

**Figure 3:**
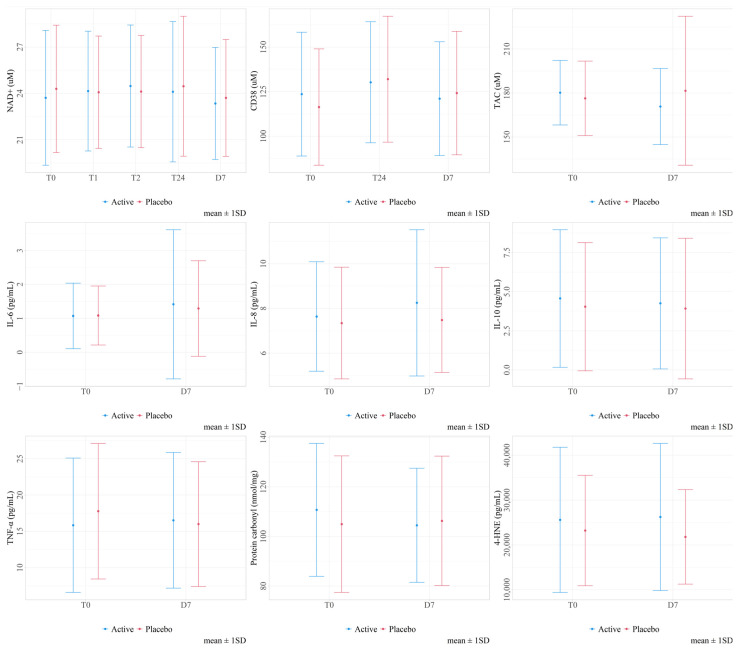
Graphs showing the means and standard deviations for each biomarker concentration for each treatment’s outcomes (combining the two treatment periods, e.g., active or placebo) across all available time points. All target analytes were measured at T0 (baseline) and at the end of each 1-week study period (D7). In addition, NAD+ was measured 1 (T1), 2 (T2), and 24 (T24) hours after the intake of the first capsule in each period. CD38 was also measured 24 h (T24) after the first capsule intake.

For each blood biomarker, the baseline concentrations (T0, period 1) are shown in Table 3, while the comparison between the treatments and different time points are shown in Table 4 and Table 5, as well as Figure 2 and Figure 3. After the adjustment for multiple testing, we did not observe any statistically significant difference in the change in any of the blood markers between the two treatment sequences. Furthermore, no difference over time was observed during the active treatment period. By contrast, during the placebo period, there was a significant increase in the blood CD38 concentrations from T0 to T24 (*p*-value = 0.019) and a significant decrease in the TNF-α concentrations between T0 and one week (*p*-value = 0.009). No other statistically significant findings were found. In the group with the active/placebo sequence, after a one-week intake of DailyColors™, there was a decreasing trend of plasma 4-HNE levels during the washout period, which continued into the placebo period. The findings remained unchanged when excluding those with high coefficients of variation (CoVs) and any outliers, after excluding the two participants who did not fast at one of the baseline assessments.

### 3.3. Outcomes: Epigenetics

**Figure 4:**
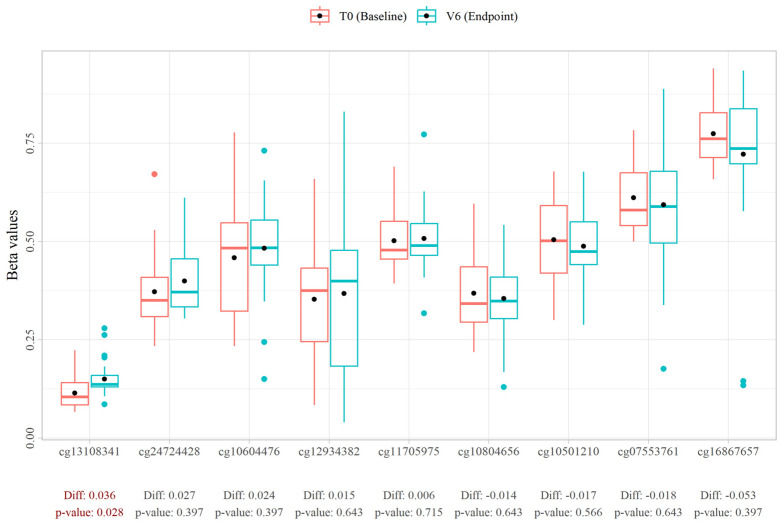
Graphs showing the beta values of each CpG island tested at T0 (baseline, red) and V6 (endpoint, blue) of this open-label study. The change in the mean beta values over the study period and respective *p*-values is reported below each CpG site.

Following this 1-month open-label study, consisting of the daily consumption of 150 mg DailyColors™ supplements, we measured DNA methylation and compared this to the DNA methylation levels at the baseline. Most candidate CpG sites remained stable (Figure 4 and Supplementary Table S2). However, interestingly, one site, cg13108341, showed hypermethylation in the post-intervention samples (0.15) when compared to the pre-intervention samples’ (0.114) CpG methylation (*p*-value = 0.021). Individual-level data are provided in Supplementary Table S3. When extending the analysis to all the CpG sites tested (~30,000 sites), rather than restricting it to the nine CpGs of interest, several CpG sites showed significant changes. We are providing details of the 27,997 sites analysed in Supplementary Table S4 to aid future investigations.

### 3.4. Safety and Tolerability

The product was relatively well tolerated, with no dropouts due to adverse events. The reported side effects are shown in Table 6. During week 1, in the group receiving the active compound, one participant reported experiencing a stomach-ache on the morning of day 2 and itchy skin throughout the week starting from day 2. Another participant in the same group reported feeling bloated throughout the entire week. In the placebo group, one participant experienced bloating and gas starting from day 3 and throughout the week. During week 2, in the group receiving the placebo, one participant had loose stools for most of the week and experienced diarrhoea on day 3. In group 2, once they were receiving the active compound, one participant reported feeling bloated during the first three days. Another participant experienced a range of side effects, including a headache, elevated temperature, nausea, and diarrhoea.

## 4. Discussion

This is a single-centre, placebo-controlled, randomised, short-term (two one-week treatment periods) cross-over intervention study to determine the effects of a supplement, DailyColors™, on the blood-based markers of mitochondrial function, oxidative stress, and inflammation. Although we did not observe any statistically significant difference in the change in any of the blood markers between the two treatment sequences, there were significant changes in two biomarkers, namely increased CD38 levels and decreased TNF-α levels, during the placebo period, which were not observed in the active period. Given the role of CD38 in ageing, NAD+ degradation, and subsequent mitochondrial dysfunction, inflammation, and oxidative stress [12,13], this could indicate a potential protective effect of the supplement against age-related biological changes. Furthermore, we conducted a one-month open-label follow-up to determine the effects of the supplementation on saliva’s DNA methylation pattern, particularly on CpG sites that have shown a tight correlation to ageing. We observed hypermethylation in one of the CpG sites. Taken together, while minimal effects were observed in this study, DailyColors™ supplementation may be beneficial by mitigating age-related changes, which should be further assessed in longer and larger scale trials of DailyColors™ supplementation. Additionally, future trials may investigate the dose response effects and safety profiles at higher doses.

DailyColors™ includes phytochemicals extracted from the plants listed in Supplementary Table S1, such as Quercetin [14], Luteolin [15], Catechins [16], Punicalagins [17], Phloretin [18], Ellagic Acid [19], Naringin [20], Apigenin [21], Isorhamnetin [22], Chlorogenic Acids [23], Rosmarinic Acid [24], Anthocyanins [25], Kaempferol [26], Proanthocyanidins [27], Myricetin [28], and Betanin [29], which have been shown to elicit antioxidant and anti-inflammatory actions in several clinical and preclinical studies [30]. Of the nine blood biomarkers measured, we observed significant alterations in CD38 (increased concentrations between T0 and T24) and TNF-α (decreased concentrations between T0 and one week) in the placebo period, which were not observed in the active period. First, the current results on CD38 concentrations are promising. CD38 is a NADase in mammalian tissue and is involved in the degradation of NAD+ [12,13]. The activity of CD38 increases with ageing and diet-induced obesity, leading to NAD+ decline [12,13]. In line with this, we observed a significant increase in CD38 concentrations among the participants (all with a BMI over 25) during the placebo period. Notably, this increase was not observed in the active period, suggesting that the DailyColors™ supplementation could have mitigated the increase in CD38 concentrations. Next, it is recognised that NAD+ decline contributes to ageing and age-related diseases through the alteration of biological mechanisms such as inflammation and oxidative stress [12,13]. In this study, besides TNF-α, we did not observe any significant changes in NAD+ or inflammatory and oxidative stress markers during both the active and placebo periods. Interestingly, we observed a decreasing trend of 4-HNE, an oxidative stress biomarker that is associated with the development of ageing-related diseases [31,32], after a one-week intake of DailyColors™. This implied that DailyColors™ supplementation may be associated with diminished oxidative stress via this biomarker. We postulate that the general lack of significant blood biomarker changes could be attributed to the very short intervention period, resulting in a minimum impact on these downstream biological mechanisms. Further studies with longer intervention and observation periods, higher doses, more participants, and proteomic analyses to determine the downstream effects of such supplementation are warranted [33]. Moreover, future in vitro and in vivo studies may examine the potential underlying mechanisms of DailyColors™ on mitochondrial function and antioxidant defence, as well as its effect on the gut microbiota composition [34]. 

Out of the nine CG sites utilised in common ageing clocks, including the Muhdo ageing clock, only cg13108341, a site with an association with the gene DNAH9, showed a significant change (*p* = 0.003). A study in 2023 analysing forensic age (age prediction) using the EPIC 850k Illumina array showed that this site has a very strong rate of change with ageing with a general hypomethylation effect [35]. Our analysis highlighted hypermethylation on the site, which is against the general trend effect of age on this site. This effect may be enhanced with higher dosages or/and increased time on the supplement. 

As cg13108341 has been highlighted as an important site for age prediction, this finding supports a potential role for DailyColors™ in reversing age-related signatures [35,36] and suggests that longer and better-powered studies might detect more relevant changes. Finally, even though several sites showed differential DNA methylation following the analysis across all the measured CpG sites, as the functions of many of these sites remain unknown, we are unable to draw further conclusions from these data. However, others have previously linked increased polyphenol levels to altered DNA methylation patterns and to a lower biological age [37,38].

There are limitations to the study design, and, thus, these results need to be interpreted cautiously. Firstly, though DailyColors™ contains many components of the MeDi diet, it does not contain any fibre, which is reported to be an important and beneficial component of this diet [34]. While previous studies have reported changes in oxidative stress- and inflammation-related biomarkers after short-term (e.g., one-week) dietary/supplement intervention [39,40], we did not observe many significant results in this current study. We postulate that the intervention duration may be too short for any significant alteration in the biological mechanisms. Future studies may consider the long-term effects of DailyColors™ on the plasma biomarkers and saliva DNA methylation. The sample size was small and based on a previous study where NAD+ was measured in red blood cells, which might be a substrate that is better suited to demonstrate the effects. The relatively low dosing, one capsule per day, which was expected to reflect the Mediterranean diet, may have been too low to show significant changes during a short period, in particular in the plasma/serum rather than blood cells. Finally, the cross-sectional design, while having benefits such as a somewhat increased statistical power and the participants being their own control with there being no risk of group differences, there is the risk of a carry-over effect. In two of the measures, IL-8 and TNF-α, there were indications that the one-week washout period might have been too short to avoid carry-over effects. We also explored the possible effects of age, BMI, and gender and repeated the analyses without outliers without significant findings. 

## 5. Conclusions

In conclusion, our study shows that DailyColors™ supplementation holds promise as a safe approach to alleviate the underlying effects of ageing. Future work is needed to determine the long-term effects of DailyColors™ supplementation on larger and more diverse cohorts.

## Figures and Tables

**Table 1:** Nine CpG sites included in this analysis and the genes affected by each site.

Sites Analysed	Gene
cg10501210	C1orf132
cg24724428	ELOVL2
cg10804656	Island (intergene)
cg10604476	ICAM5
cg11705975	PRLHR
cg12934382	GRM2
cg07553761	TRIM59
cg16867657	ELOVL2
cg13108341	DNAH9

**Table 2:** Baseline characteristics of study participants.

	Active/Placebo	Placebo/Active	Total
Sex			
Female	7 (46.7)	9 (60.0)	16 (53.3)
Male	8 (53.3)	6 (40.0)	14 (46.7)
Age, years	66.5 (8.2)	67.5 (6.9)	67.0 (7.5)
BMI, kg/m^2^	29.2 (3.4)	28.2 (2.5)	28.7 (3.0)
HbA1c, mmol/mol	38.4 (3.5)	41.7 (8.5)	40.0 (6.6)
Glucose, mmol/L	6.4 (1.6)	6.4 (1.9)	6.4 (1.7)
Cholesterol			
HDL, mmol/L	1.3 (0.4)	1.4 (0.3)	1.4 (0.4)
LDL, mmol/L	3.8 (1.5)	4.0 (1.2)	3.9 (1.4)

Mean and standard deviation for quantitative variables and frequency and percentage for sex.

**Table 3:** Baseline concentrations of the blood biomarkers.

	Active/Placebo	Placebo/Active	Total
NAD+ (µM)	23.8 (5.2)	25.5 (4.9)	24.6 (5.0)
CD38 (µM)	128.7 (41.3)	110.5 (32.4)	119.6 (37.6)
TAC (µM)	181.5 (28.6)	175.0 (28.0)	178.3 (28.0)
IL-6 (pg/mL)	1.3 (1.0)	0.8 (0.7)	1.0 (0.9)
IL-8 (pg/mL)	8.5 (2.2)	7.1 (2.7)	7.8 (2.5)
IL-10 (pg/mL)	6.3 (4.4)	2.9 (3.7)	4.6 (4.3)
TNF-α (pg/mL)	20.4 (10.3)	16.8 (7.1)	18.6 (8.9)
Protein carbonyl (nmol/mg)	103.8 (30.5)	108.6 (32.2)	106.2 (30.9)
4-HNE (pg/mL)	27,367.1 (19,872.1)	20,620.1 (8292.1)	23,993.6 (15,349.5)

Mean and standard deviation for baseline concentrations of blood biomarkers.

**Table 4:** Concentrations at baseline, at one week, and differences for each outcome.

		Baseline	At One Week	Difference	*p*-Value	Adj. *p*-Value
Main outcomes						
NAD+	Active	23.7 (4.4)	23.3 (3.6)	0.4 (3.8)	0.600	0.936
	Placebo	24.3 (4.1)	23.7 (3.8)	0.6 (3.1)	0.309	0.936
CD38	Active	123.6 (34.8)	121.1 (31.9)	2.6 (27.2)	0.610	0.857
	Placebo	116.4 (32.7)	124.2 (34.7)	−7.8 (20.6)	**0.046**	0.215
Secondary outcomes						
TAC	Active	180.2 (22.0)	170.7 (25.9)	9.5 (28.1)	0.076	0.218
	Placebo	176.4 (25.4)	181.5 (50.8)	−5.2 (50.0)	0.576	0.849
IL-6	Active	1.1 (1.0)	1.4 (2.2)	−0.3 (2.1)	0.368	0.611
	Placebo	1.1 (0.9)	1.3 (1.4)	−0.2 (1.6)	0.479	0.611
IL-8	Active	7.6 (2.4)	8.3 (3.3)	−0.6 (3.1)	0.282	0.645
	Placebo	7.3 (2.5)	7.5 (2.4)	−0.1 (2.0)	0.711	0.467
IL-10	Active	4.6 (4.4)	4.3 (4.2)	0.3 (2.6)	0.510	0.223
	Placebo	4 (4.1)	3.9 (4.5)	0.1 (1.3)	0.606	0.223
TNF-α	Active	15.8 (9.3)	16.5 (9.3)	−0.7 (4.8)	0.443	0.435
	Placebo	17.8 (9.3)	16 (8.6)	1.8 (5.2)	0.073	**0.009**
Protein carbonyl	Active	110.7 (26.8)	104.5 (23.0)	6.2 (33.3)	0.315	0.966
	Placebo	104.9 (27.5)	106.3 (26.1)	−1.3 (26.6)	0.787	0.636
4-HNE	Active	25,593.8 (16,185.3)	26,231.7 (16,413.5)	−637.9 (7937.9)	0.663	0.074
	Placebo	23,210.5 (12,309.6)	21,791.1 (10,535.9)	1419.3 (10,453.7)	0.463	0.299

The adjusted *p*-values were calculated by considering the period and carry-over and correcting for multiple testing with the false discovery rate (FDR). The body mass index (BMI) was significant in IL-6, and sex was significant in TNF-α. The period was significant in NAD+ (*p* = 0.097), IL-8 (*p* = 0.033), and 4-HNE (*p* = 0.018). The carry-over was significant in IL-8 (*p* = 0.008), IL-10 (*p* = 0.076), TNF-α (*p* = 0.008), and 4-HNE (*p* = 0.002). *p*-values <0.05 shown in bold.

**Table 5:** Differences for each outcome at one hour, two hours, and twenty-four hours compared to baseline.

		NAD+		CD38	
Time	Treatment	Difference	*p*-Value	Difference	*p*-Value
T0 vs. T1	Active	−0.50 (0.6)	0.936	-	-
T0 vs. T2	Active	−0.82 (0.6)	0.936	-	-
T0 vs. T24	Active	−0.32 (0.6)	0.936	−8.47 (5.4)	0.215
T0 vs. T1	Placebo	0.19 (0.6)	0.943	-	-
T0 vs. T2	Placebo	0.16 (0.6)	0.943	-	-
T0 vs. T24	Placebo	−0.06 (0.6)	0.963	−16.88 (5.4)	**0.019**

The models were fitted using logarithm transformations. The results are presented to the original scale. The results are presented as the estimated difference and standard error. False discovery rate (FDR) correction for multiple testing was applied. The results are the first measurement (e.g., the baseline)–the follow-up, e.g., negative values mean an increased concentration. The follow-up time points include 1 h (T1), 2 h (T2), and 24 h (T24) after the intake of the first capsule in each period. *p*-values <0.05 shown in bold.

**Table 6:** Side effects.

Sequence	Side Effects Week 1	Side Effects Week 2
Active/Placebo	Bloated all week, stomach-ache morning of day 2, itchy skin every day from day 2 (cold with a temperature on days 4 and 5)	Loose stools, diarrhoea
Placebo/Active	Bloating, heartburn	Bloating, headache, temperature, nausea, diarrhoea

## Data Availability

The data that support the findings of this study are available from the corresponding authors, C.d.L. and J.R.C., upon reasonable request.

## Supplementary Materials

The following supporting information can be downloaded at https://www.mdpi.com/article/10.3390/antiox13080995/s1. Table S1: the investigational product; Table S2: the mean, minimum 25th percentile, 75th percentile, and maximum values for each candidate CpGs site at T0 (the baseline) and V6 (the endpoint); Table S3: the beta values of each of the nine candidate CpG islands tested at T0 (the baseline) and V6 (the endpoint) for each subject; and Table S4: the mean, minimum, 5th percentile, 25th percentile, 75th percentile, 95th percentile, and maximum values at T0 (the baseline) and V6 (the baseline) and the change in mean values between 6 and T0 for the 27997 CG sites showing the most significant change (all sites up to *p* = 1 are included).
