# Supplementary material for: A Randomised, Double-Blind, Placebo-Controlled, Cross-Over Clinical Trial to Evaluate the Biological Effects and Safety of a Polyphenol Supplement on Healthy Ageing
Source: Antioxidants (Basel). 2024 Aug 17;13(8):995. doi: 10.3390/antiox13080995 (PMC11352085; doi:10.3390/antiox13080995)

Supplementary material

Supplementary Table S1. The investigational product

DailyColors™ capsules contain 150mg of the following phytonutrients:

Item Name	Per cent of Formula
Apple Extract	10.0%
Pomegranate Extract	10.0%
Tomato Powder	2.5%
Beet - Spray Dried	2.5%
Olive Extract	7.5%
Rosemary Extract	7.5%
Green Coffee Bean Extract (CA)	7.5%
Kale - Freeze Dried	2.5%
Onion Extract	10.0%
Ginger Extract	10.0%
Grapefruit Extract	2.5%
Carrot - Air Dried	2.5%
Grape Skin Extract	17.5%
Blueberry Extract	2.5%
Current - Freeze Dried	2.5%
Elderberry - Freeze Dried	2.5%

Placebo content
Microcrystalline cellulose was used in the placebo capsule, produced with a similar appearance as the active compound visually and regarding smell and taste.

List of phytochemicals as per Daily Colors US Patent Application (Serial. No. 17/843,140)

Phytochemicals
Quercetin
Luteolin
Catechins
Punicalagins
Phloretin
Ellagic Acid
Naringin
Apigenin
Isorhamnetin
Chlorogenic Acids
Rosmarinic Acid
Anthocyanins
Kaempferol
Proanthocyanidins
Myricetin
Betanin

Supplementary Table S2. Mean, standard deviation, minimum, 25th percentile, 75th percentile, and maximum values for each candidate CpGs site at T0 (baseline) and V6 (endpoint)

CpG Name	T0 (Baseline)							V6 (Endpoint)							Mean Change V6 - T0
	Mean	Standard Deviation	Minimum	25th percentile	Median	75th percentile	Maximum	Mean	Standard Deviation	Minimum	25th percentile	Median	75th percentile	Maximum	
cg13108341	0.114	0.038	0.067	0.084	0.105	0.141	0.223	0.150	0.045	0.086	0.130	0.136	0.160	0.279	0.036
cg24724428	0.372	0.093	0.234	0.309	0.350	0.409	0.671	0.400	0.086	0.304	0.334	0.372	0.456	0.611	0.027
cg10604476	0.458	0.156	0.234	0.323	0.484	0.548	0.777	0.483	0.125	0.150	0.440	0.484	0.554	0.731	0.024
cg12934382	0.353	0.149	0.084	0.245	0.376	0.432	0.659	0.368	0.204	0.040	0.183	0.400	0.478	0.830	0.015
cg11705975	0.502	0.074	0.393	0.455	0.478	0.552	0.690	0.507	0.089	0.317	0.465	0.490	0.546	0.772	0.006
cg10804656	0.368	0.103	0.219	0.295	0.342	0.436	0.596	0.355	0.097	0.130	0.304	0.348	0.410	0.542	-0.014
cg10501210	0.504	0.104	0.300	0.420	0.502	0.591	0.678	0.488	0.091	0.288	0.441	0.474	0.550	0.677	-0.017
cg07553761	0.611	0.089	0.500	0.541	0.580	0.675	0.783	0.593	0.167	0.176	0.496	0.589	0.678	0.888	-0.018
cg16867657	0.774	0.079	0.658	0.714	0.761	0.827	0.940	0.722	0.192	0.134	0.698	0.736	0.837	0.934	-0.053

Supplementary Table S3. Beta values of each of the 9 candidate CpG island tested at T0 (baseline) and V6 (endpoint), for each subject

Subject	Time	cg07553761	cg10501210	cg10604476	cg10804656	cg11705975	cg12934382	cg13108341	cg16867657	cg24724428
1	T0	0.535	0.426	0.438	0.367	0.407	0.459	0.099	0.685	0.267
1	V6	0.382	0.453	0.472	0.261	0.425	0.484	0.138	0.651	0.305
2	T0	0.566	0.576	0.238	0.317	0.453	0.084	0.067	0.769	0.402
2	V6	0.769	0.634	0.244	0.168	0.542	0.040	0.106	0.899	0.458
3	T0	0.773	0.578	0.478	0.494	0.630	0.405	0.117	0.738	0.472
3	V6	0.864	0.523	0.442	0.542	0.627	0.313	0.262	0.864	0.576
4	T0	0.611	0.461	0.489	0.487	0.416	0.349	0.171	0.661	0.305
4	V6	0.500	0.575	0.511	0.384	0.429	0.413	0.134	0.661	0.345
5	T0	0.508	0.426	0.290	0.253	0.413	0.106	0.174	0.684	0.324
5	V6	0.470	0.349	0.444	0.307	0.317	0.156	0.156	0.577	0.316
6	T0	0.500	0.624	0.694	0.293	0.472	0.377	0.136	0.817	0.275
6	V6	0.338	0.559	0.637	0.282	0.462	0.443	0.145	0.145	0.364
7	T0	0.674	0.485	0.378	0.322	0.489	0.361	0.087	0.760	0.362
7	V6	0.590	0.439	0.458	0.352	0.554	0.404	0.086	0.673	0.319
8	T0	0.540	0.418	0.402	0.352	0.467	0.433	0.143	0.762	0.290
8	V6	0.566	0.475	0.395	0.358	0.481	0.370	0.134	0.784	0.402
9	T0	0.675	0.460	0.777	0.244	0.509	0.416	0.143	0.874	0.350
9	V6	0.446	0.442	0.731	0.385	0.413	0.533	0.141	0.708	0.351
10	T0	0.504	0.400	0.504	0.287	0.472	0.490	0.067	0.718	0.303
10	V6	0.483	0.420	0.483	0.130	0.408	0.519	0.130	0.731	0.306
11	T0	0.683	0.614	0.234	0.451	0.566	0.199	0.085	0.873	0.529
11	V6	0.617	0.520	0.459	0.522	0.513	0.301	0.144	0.846	0.522
12	T0	0.783	0.595	0.267	0.596	0.690	0.112	0.120	0.940	0.671
12	V6	0.717	0.511	0.395	0.473	0.626	0.131	0.131	0.934	0.471
13	T0	0.592	0.549	0.584	0.402	0.544	0.426	0.078	0.756	0.409
13	V6	0.695	0.487	0.562	0.332	0.536	0.695	0.134	0.134	0.459
14	T0	0.543	0.300	0.319	0.219	0.524	0.230	0.101	0.790	0.370
14	V6	0.176	0.395	0.501	0.285	0.488	0.176	0.165	0.839	0.379
15	T0	0.706	0.402	0.607	0.295	0.554	0.659	0.147	0.837	0.408
15	V6	0.629	0.474	0.610	0.377	0.476	0.577	0.143	0.739	0.352
16	T0	0.565	0.331	0.500	0.402	0.472	0.374	0.080	0.712	0.297
16	V6	0.585	0.288	0.548	0.325	0.491	0.407	0.161	0.696	0.319
17	T0	0.611	0.580	0.470	0.320	0.533	0.362	0.083	0.755	0.351
17	V6	0.793	0.677	0.439	0.439	0.611	0.121	0.135	0.864	0.611
18	T0	0.732	0.678	0.535	0.523	0.599	0.582	0.223	0.869	0.378
18	V6	0.552	0.472	0.555	0.303	0.527	0.649	0.182	0.820	0.362
19	T0	0.568	0.595	0.249	0.310	0.461	0.144	0.100	0.786	0.345
19	V6	0.598	0.573	0.362	0.306	0.484	0.081	0.279	0.799	0.339
20	T0	0.768	0.645	0.497	0.390	0.485	0.291	0.130	0.924	0.482
20	V6	0.621	0.445	0.604	0.417	0.547	0.395	0.110	0.811	0.451
21	T0	0.613	0.549	0.336	0.388	0.559	0.166	0.084	0.831	0.441
21	V6	0.888	0.600	0.347	0.291	0.772	0.069	0.210	0.888	0.451
22	T0	0.518	0.473	0.502	0.332	0.446	0.418	0.117	0.658	0.320
22	V6	0.495	0.441	0.552	0.317	0.449	0.443	0.111	0.702	0.332
23	T0	0.533	0.414	0.552	0.295	0.467	0.525	0.097	0.728	0.336
23	V6	0.588	0.486	0.485	0.466	0.503	0.830	0.117	0.728	0.383
24	T0	0.545	0.629	0.687	0.231	0.393	0.444	0.109	0.703	0.234
24	V6	0.879	0.618	0.510	0.345	0.561	0.204	0.205	0.703	0.402
25	T0	0.561	0.383	0.652	0.447	0.434	0.431	0.074	0.692	0.339
25	V6	0.558	0.361	0.655	0.467	0.474	0.459	0.119	0.734	0.304
26	T0	0.675	0.519	0.240	0.564	0.592	0.329	0.143	0.806	0.418
26	V6	0.627	0.461	0.150	0.387	0.479	0.351	0.131	0.833	0.510

Supplementary Figure S1. Flow diagram illustrating the study design

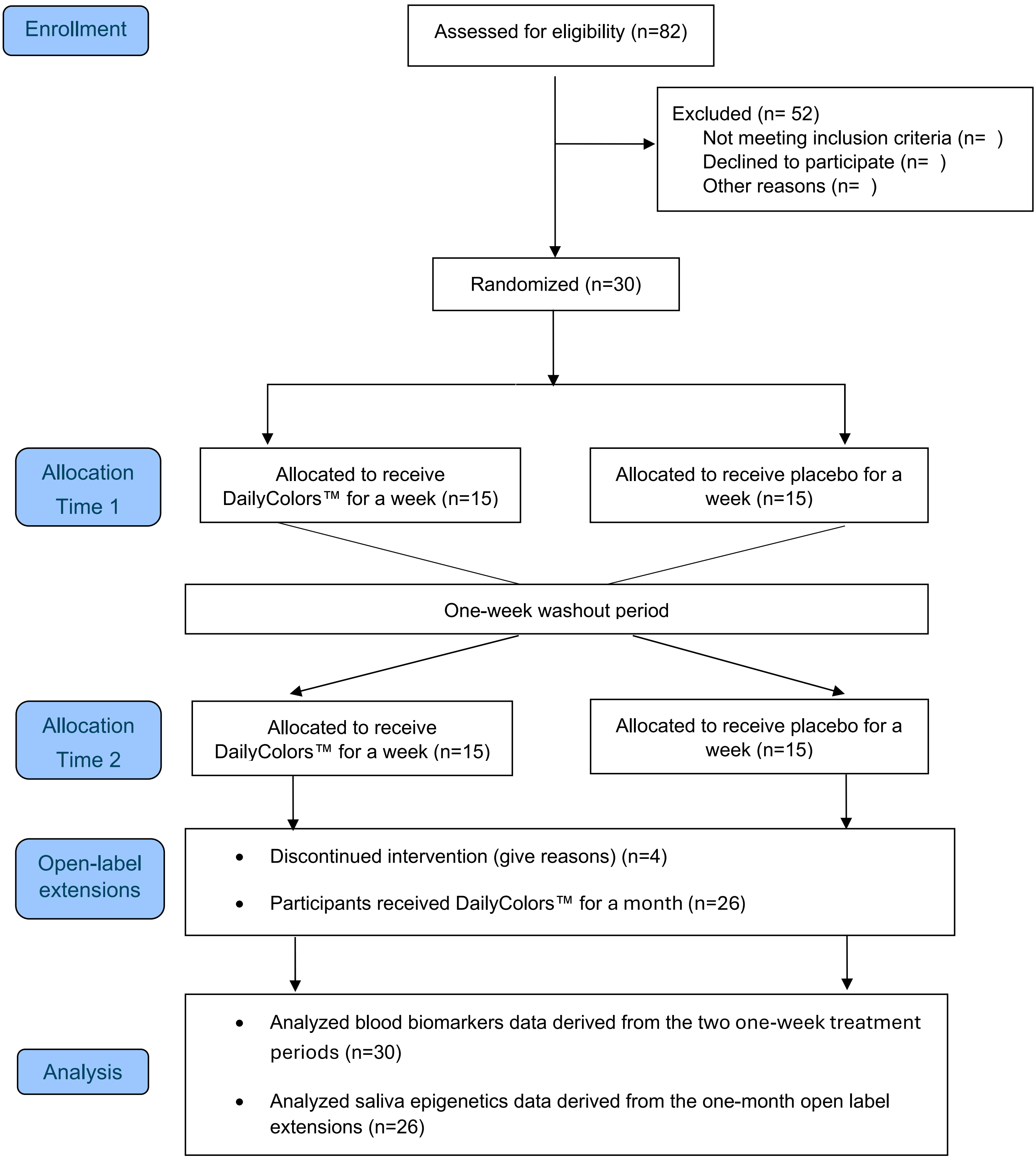

Supplement: Supplementary file 1 [file antioxidants-13-00995-s001.zip › antioxidants-3082742-supplementary.pdf]
